# Chimeric viruses between Rocio and West Nile: the role for Rocio prM-E proteins in virulence and inhibition of interferon-α/β signaling

**DOI:** 10.1038/srep44642

**Published:** 2017-03-20

**Authors:** Alberto A. Amarilla, Yin X. Setoh, Parthiban Periasamy, Nias Y. Peng, Gabor Pali, Luiz T. Figueiredo, Alexander A. Khromykh, Victor H. Aquino

**Affiliations:** 1 Laboratory of Virology, Department of Clinical Analyses, Toxicology and Food Sciences, School of Pharmaceutical Sciences of Ribeirao Preto, University of Sao Paulo, Ribeirao Preto, Sao Paulo, Brazil; 2 Australian Infectious Diseases Research Centre, School of Chemistry and Molecular Biosciences, The University of Queensland, St Lucia, Queensland, 4072, QLD, Australia; 3 Virology Research Center, School of Medicine of Ribeirao Preto, University of de Sao Paulo, Ribeirao Preto, Sao Paulo, Brazil

## Abstract

Mosquito-transmitted flavivirus Rocio (ROCV) was responsible for an outbreak of encephalitis in the Ribeira Valley, located in the south coast of Sao Paulo State, Brazil, in 1975–1976. ROCV also causes fatal encephalitis in adult mice. Seroprevalence studies in humans, horses and water buffaloes in different regions of Brazil have suggested that ROCV is still circulating in the country, indicating the risk of re-emergence of this virus. West Nile virus (WNV) is also a mosquito-transmitted encephalitic flavivirus, however, WNV strains circulating in Australia have not been associated with outbreaks of disease in humans and exhibit low virulence in adult mice. To identify viral determinants of ROCV virulence, we have generated reciprocal chimeric viruses between ROCV and the Australian strain of WNV by swapping structural prM and E genes. Chimeric WNV containing ROCV prM-E genes replicated more efficiently than WNV or chimeric ROCV containing WNV prM-E genes in mammalian cells, was as virulent as ROCV in adult mice, and inhibited type I IFN signaling as efficiently as ROCV. The results show that ROCV prM and E proteins are major virulence determinants and identify unexpected function of these proteins in inhibition of type I interferon response.

*Rocio virus* (ROCV) is a member of the genus *Flavivirus* in the family *Flaviviridae*. Like other members of the family *Flaviviridae*, ROCV is an enveloped virus containing positive-sense RNA genome of approximately 11 kb^1^. The viral RNA encodes a single open reading frame (ORF) that is flanked by untranslated regions at the 5′ and 3′ ends (5′UTR and 3′UTR). The ORF encodes a single polyprotein, which is co- and post-translationally cleaved into 3 structural (C, prM and E) and 7 nonstructural proteins (NS1-NS2A-NS2B-NS3-NS4A-NS4B-NS5)^2^. Phylogenetic analysis suggests that ROCV is closely related to the members of *Japanese encephalitis virus* (JEV) serogroup, which also includes *West Nile virus* (WNV)^3^. ROCV was isolated from a pool of mosquitoes of the *Psorophora ferox* species, confirming that it is transmitted by mosquitoes^4^.

ROCV was responsible for an outbreak of encephalitis which occurred in 1975 in the Ribeira Valley, located at the southern coast of Sao Paulo State, Brazil^5^. A total of 465 cases of encephalitis with 61 deaths (13% case fatality rate) were reported from that outbreak^6^. From 1976, one year after the outbreak, ROCV cases subsided, however seroprevalence studies in humans, horses and water buffaloes in different regions of Brazil have suggested that ROCV maintains circulation across the country, indicating the risk of re-emergence of this highly virulent virus^7^,^8^,^9^,^10^,^11^.

The clinical manifestations presented by patients during the outbreak included fever, malaise, lassitude, vomit, lethargy, severe headache, dizziness, photophobia, lower-extremity weakness, and severe neurologic disorders. Neuropsychiatric sequelae were observed in 20% of the patients. Permanent sequelae included variable degrees of paralysis and other cerebral and cerebellar dysfunctions^6^,^12^. Autopsies of eight fatal cases of encephalitis caused by ROCV virus revealed histopathologic lesions in the brain with interstitial mononuclear infiltration, microglial proliferation and perivascular lymphocytic cuffing^13^. Recently, evaluation of the inflammatory process caused by ROCV in an animal model has shown a significant increase of pro-inflammatory cytokines such as interferon alpha (IFN-α), interleukine-1 beta (IL1-β) and tumor necrosis factor alpha (TNF-α) in the central nervous system (CNS), as well as the presence of inflammatory cell, neuronal degeneration and apoptosis^14^. The type I interferons (IFN-α/β) are the major cytokines produced in response to viral infections that are responsible for the activation of a complex signaling cascade, leading to regulation of the expression of several IFN-stimulated genes (ISGs) such as IL1-β and TNF-α, among others, to fight viral infections^15^,^16^. The roles of IFN-α/β as antiviral molecules have been widely studied^17^. The virus’ ability to block or escape the IFN-α/β response may determine viral pathogenicity^18^. Inhibition of IFN-α/β signaling by nonstructural proteins of flaviviruses such as *Dengue virus* (DENV), JEV, *Tick borne encephalitis virus* (TBEV), and WNV and its contribution to virulence has been previously demonstrated^19^,^20^,^21^,^22^. The American WNV_NY99_ strain was shown to be more virulent in mice than the Australian Kunjin virus (KUNV), a naturally attenuated subtype of WNV, and this was attributed to the more efficient inhibition of type I IFN response mediated by non-structural proteins, including NS55^20^,^23^,^24^. On the other hand, an attenuated JEV vaccine candidate was shown to have reduced ability to block the IFN-α/β signaling that was caused by a single E138K mutation in the structural E protein^25^.

Recently, we have described a simple and efficient method for generation of WNV infectious cDNAs, termed circular polymerase extension cloning (CPEC), and used it to produce chimeric viruses between attenuated Australian and virulent American WNV strains in order to identify viral genetic determinants of virulence^26^,^27^,^28^. In the current study, we employed the CPEC method to generate infectious cDNA of ROCV and reciprocal prM-E chimeric viruses between ROCV and Australian strain of WNV. We employed these chimeric viruses to show that ROCV prM-E proteins are the major contributors to the virulence of ROCV and identified unexpected role of ROCV prM-E proteins in inhibition of IFN-α/β signaling.

## Results

### Construction of the full-length infectious cDNA of ROCV by CPEC

**Figure 1:**
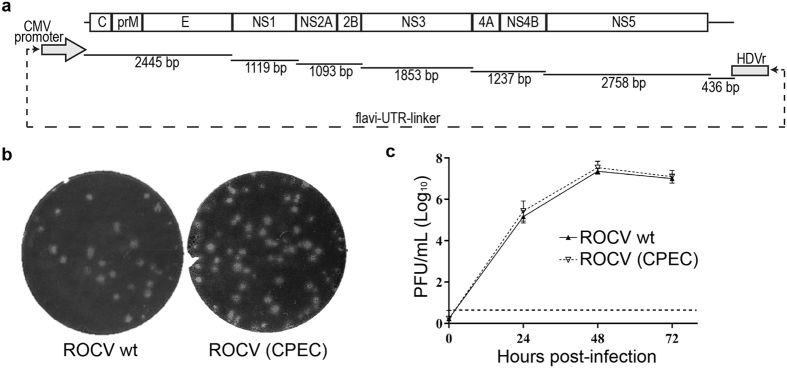
Generation by CPEC and characterization of ROCV. (**a**) The strategy for constructing the full-length infectious cDNA of ROCV by CPEC reaction. (**b**) Plaque morphology of parental and CPEC-generated ROCV (passage 1) in BHK-21 cells. (**c**) Replication efficiencies of parental and CPEC-generated ROCV in mouse embryonic fibroblasts (MEF). Cells were infected at MOI = 0.1 with parental or CPEC-generated ROCV, culture supernatant were collected at 0, 24, 48 and 72 hours post-infection, and viral titers were determined by plaque assay on BHK-21 cells. The dashed line represents the LOD of the assay.

To generate infectious cDNA of ROCV, viral RNA was purified from the supernatant of C6/36 cell culture infected with ROCV (SPH 34675 strain) and used to generate 7 cDNA fragments spanning the complete viral genome by RT-PCR (Fig. 1a). Equimolar amounts of the 7 RT-PCR fragments and a flavi-UTR-linker fragment containing CMV promoter, conserved first and last 22 nucleotides of viral sequence and hepatitis delta virus ribozyme^26^, were used in CPEC reaction (Fig. 1a, Supplementary Table S1). The circular DNA product obtained in the CPEC reaction was transfected into HEK293T cells, which were incubated for five days before collecting virus-containing culture fluid (passage 0, p0). Recovered virus was then passaged once in HEK293T cells (p1) and compared with wt ROCV isolate. Both viruses produced plaques of similar size and morphology in BHK cells (Fig. 1b), and replicated with similar efficiencies in mouse embryonic fibroblasts (MEF) (Fig. 1c). These results demonstrate that ROCV was successfully recovered using CPEC, and the CPEC-generated ROCV is indistinguishable from the parental virus. The recovered ROCV and both chimeric viruses (all passage 1 stock) were sequenced using Illumina Nextera XT. Sequencing results showed a number of changes from previously published ROCV sequence that were present in the recovered ROCV and also in the corresponding ROCV regions in respective chimeric viruses (denoted in green in Supplementary Table S2). These are likely to be either mistakes in the previous sequence of ROCV deposited to GenBank^3^ or changes accumulated during passaging of the ROCV isolate used in this study. We have previously shown that CPEC methodology allows recovery of a virus that accurately represents the original virus population^27^; thus deep sequencing of the ROCV isolate used for CPEC recovery was deemed unnecessary. The only other differences identified by sequencing were mixed population of Ala and Ser residues at position 111 in the C gene and a mixed population of T and C nucleotides in Ala residue at position 66 in the NS2A gene (not causing amino acid change) in the ROCV/WNV-prME chimeric virus compared to the recovered ROCV (denoted in red in Supplementary Table S2). A mixed population of Asn and Ser residues at position 89 in the E gene was also identified in both the recovered ROCV and WNV/ROCV-prME chimeric virus with Ser being more prominent in both viruses (denoted in blue in Supplementary Table S2).

### ROCV prM-E proteins confer advantage in replication in mammalian cells

**Figure 2:**
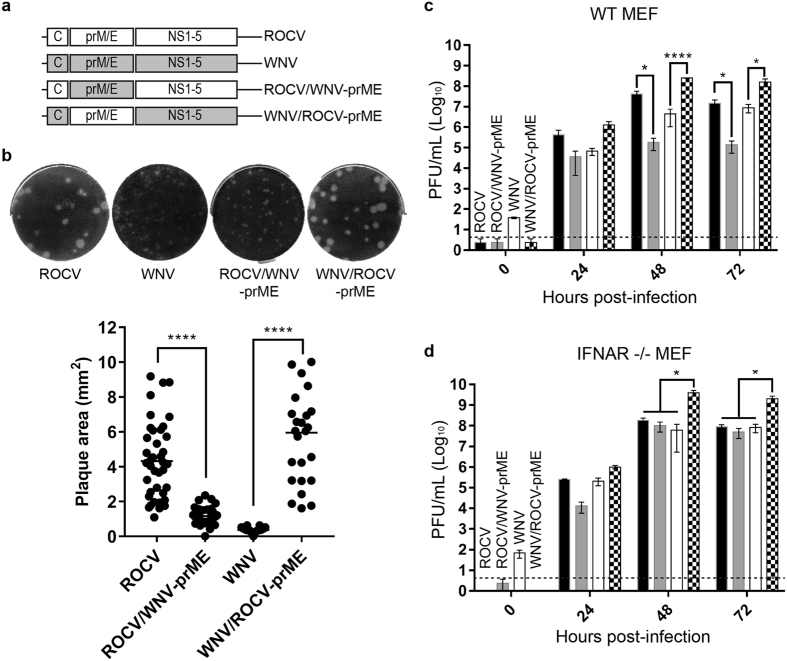
Construction and characterization of ROCV/WNV chimeric viruses. (**a**) Schematic representation of ROCV/WNV_NSW2011_ chimeric viruses. (**b**) Plaque morphology of parental (ROCV and WNV) and chimeric (ROCV/WNV-prME and WNV/ROCV-prME) viruses in BHK-21 cells (upper) and comparison of plaque size areas (lower). Plaque areas (mm^2^) for 25–40 randomly selected plaques were measured using ImageJ software (National Institutes of Health, USA) and plotted as mean ± SD. (**c**) Growth kinetics of parental and chimeric viruses in IFN-competent mouse embryonic fibroblasts (WT MEF) and (**d**) IFNAR^−/−^ MEF cells infected at MOI = 0.1. Viral titers were determined by plaque assay on BHK-21 cells at the indicated time points. The dashed lines represent the LOD of the assay. Parametric one-way ANOVA test was used to compare within groups. *P-value ≤ 0.05, ****P-value ≤ 0.0001.

We have shown previously that American WNV_NY99_ strain was more virulent than the Australian WNV_NSW2011_ strain in mice and mapped the nonstructural proteins of WNV_NY99_ strain as major contributors to increased virulence through their ability to more efficiently inhibit IFN-α/β response^26^,^29^. To analyze the potential role of ROCV proteins in virulence, we constructed two reciprocal chimeric viruses between ROCV and WNV_NSW2011_ using CPEC. WNV_NSW2011_ was chosen for generating chimeric viruses as it also belongs to the JEV serogroup of encephalitis flaviviruses and is less virulent in adult mice. The first chimeric virus, ROCV/WNV-prME, contains prM-E genes of WNV_NSW2011_ on the backbone of ROCV. The second chimeric virus, WNV/ROCV-prME, contains prM-E genes of ROCV on the backbone of WNV_NSW2011_ (Fig. 2a). Plaque assays of collected culture fluids from CPEC-transfected HEK293 cells on BHK cells showed that both chimeric viruses were successfully generated (Fig. 2b). Sequencing of the C-prM and E-NS1 junctions confirmed the correct sequences in generated chimeric viruses (data not shown). Interestingly, WNV/ROCV-prME chimeric virus produced larger plaques than parental WNV_NSW2011_, and even than the parental ROCV in BHK cells (Fig. 2b). In contrast, ROCV/WNV-prME chimeric virus produced plaques smaller than parental ROCV, and similar to those produced by WNV_NSW2011_ (Fig. 2b). Replicative efficiencies of chimeric and parental viruses were then compared in IFN-α/β response-competent (WT) or IFN-α/β response–deficient (IFNAR^−/−^) mouse embryonic fibroblasts (MEF). In WT MEF cells, ROCV/WNV-prME chimeric virus replicated less efficiently than parental ROCV, while WNV/ROCV-prME chimeric virus replicated more efficiently than parental WNV_NSW2011_ (Fig. 2c), suggesting that the ROCV prME proteins would be involved in inhibition of the IFN-α/β response. This was further supported by the similar replication efficiencies of ROCV and ROCV/WNV-prME chimeric virus in IFNAR^−/−^ MEF (Fig. 2d). However, WNV/ROCV-prME chimeric virus replicated significantly more efficiently than all other viruses in IFNAR^−/−^ MEF (Fig. 2d), indicating that prME of ROCV may also enhance virus replication independently of the inhibition of IFN-α/β response (Fig. 2d). Importantly, unlike the results in WT MEF, ROCV/WNV-prME chimeric virus reached similar to the parental ROCV titres at 48 h and 72 h after infection in IFNAR^−/−^ MEF (Fig. 2d) indicating that chimerization did not avertedly affect replication of this chimeric virus in the absence of functional IFN-α/β response.

### ROCV prM-E proteins inhibit JAK/STAT signaling

**Figure 3:**
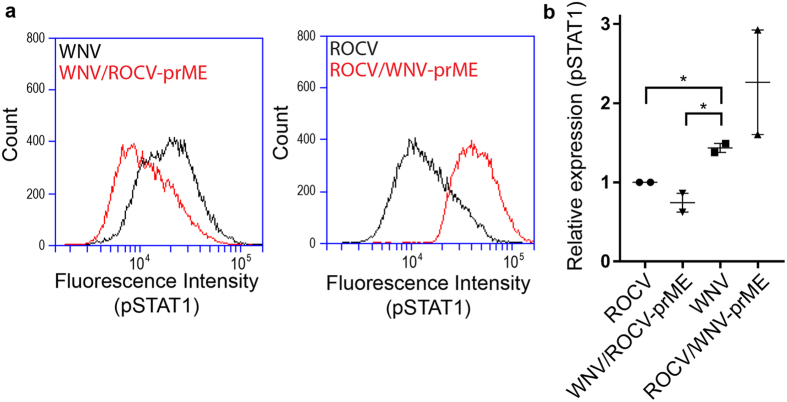
pSTAT1 analysis in IRF3^−/−^ × IRF7^−/−^ MEF cells infected with parental and chimeric viruses and treated with mouse IFN-β. (**a**) IRF3^−/−^ × IRF7^−/−^ MEF cells were infected with parental or chimeric viruses at MOI = 0.1, and treated with 10,000 IU of mouse IFN-β at 48 h.p.i. Cells were gated to the single-cell infected population, and cell counts were plotted to fluorescence intensity of pSTAT1. (**b**) Relative expression of pSTAT1 normalized to ROCV. Data represent the mean of two independent experiments; each performed in duplicate. Parametric one-way ANOVA test was used to compare within groups. *P-value ≤ 0.05.

To further investigate downstream mechanisms of inhibition of the IFN-α/β response by ROCV prM-E proteins, we used IRF3^−/−^ × IRF7^−/−^ MEF cells, which lack the ability to produce IFN-α/β in response to infection, but maintain the ability to respond to exogenous IFN-α/β. IRF3^−/−^ × IRF7^−/−^ MEF were infected with the parental and chimeric viruses at MOI = 0.1 for 48 h and then treated with 10,000 IU of mouse IFN-β for 30 min. Cells were collected and analyzed by flow cytometry by first gating to a single cell population, and then to infected cells. The intensity of pSTAT1 staining was then analyzed for the population of infected cells (Fig. 3a). The phosphorylation of STAT1 was inhibited in cells infected with ROCV and WNV/ROCV-prME, but not in cells infected with WNV_NSW2011_ or ROCV/WNV-prME (Fig. 3a). Results from two independent experiments demonstrated that ROCV- and WNV/ROCV-prME-infected cells consistently showed reduced pSTAT1 expression in response to IFN-β treatment compared to WNV_NSW2011_ -infected cells (Fig. 3b). The results show that ROCV prM-E proteins are likely to be responsible for the inhibition of JAK-STAT signaling.

### ROCV prM-E proteins provide major contribution to virulence in mice

**Figure 4:**
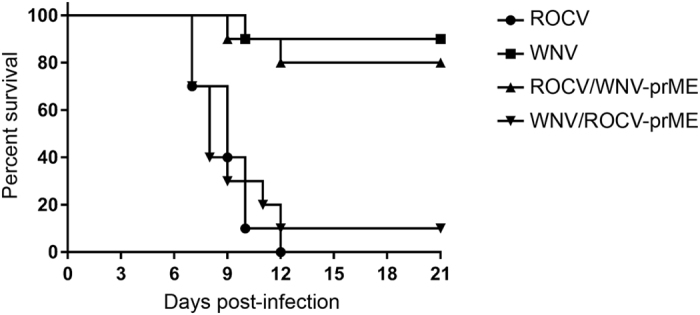
Survival of 6-week-old C57BL6 female mice after infection with parental and chimeric viruses. Six-week-old C57BL6 mice in groups of 10 were infected with 2.6 × 10^4^ pfu of the indicated viruses via the intraperitoneal route and monitored for signs of encephalitis for 21 days post-infection.

To investigate if the inhibition of JAK-STAT signaling by ROCV prM-E and enhanced replication of WNV/ROCV-prME chimeric virus in MEF also result in increased virulence *in vivo*, we infected groups of ten, 6-week-old C57BL6 female mice with 2.6 × 10^4^ pfu of parental or chimeric viruses via the intraperitoneal route. Mice were monitored for 21 days for signs of encephalitis, at which point the animals were immediately sacrificed. Infection with WNV_NSW2011_ or ROCV/WNV-prME viruses resulted in 10% and 20% mortality, respectively (Fig. 4). In contrast, infection with ROCV resulted in 100% mortality, while infection with WNV/ROCV-prME chimeric virus resulted in 90% mortality (Fig. 4). Statistical significance was observed for ROCV v.s. ROCV/WNV-prME (p ≤ 0.0001) and for WNV_NSW2011_ v.s. WNV/ROCV-prME (p ≤ 0.001). The results clearly demonstrate that ROCV prM-E proteins provide major contribution to the virus virulence in adult immunocompetent mice.

## Discussion

We have successfully generated infectious cDNA of ROCV and of chimeric viruses between ROCV and WNV using CPEC method. We have used this method previously for construction of infectious cDNAs of WNV and of chimeric viruses between Australian and American strains of WNV to identify genetic determinants involved on virulence^26^,^27^. The chimeric ROCV/WNV viruses generated in this study contained prM-E genes swapped between WNV and ROCV with the remaining genomic sequences left intact. ROCV prM-E differ from WNV_NSW2011_ prM-E by 248 from a total of 668 amino acids (62.8% homology). Previous studies with chimeric flaviviruses demonstrated that prM-E genes can be successfully exchanged, even between distantly related flaviviruses^30^,^31^,^32^,^33^,^34^; thus the successful recovery of ROCV/WNV_NSW2011_ chimeric viruses despite substantial amino acid differences was not entirely unexpected. We showed that chimeric ROCV containing WNV prM-E genes in comparison with the parental ROCV exhibited significantly reduced replication in IFN-α/β response-competent cells, decreased efficiency in inhibition of JAK-STAT signaling and lower virulence in mice. In agreement with these findings, the reciprocal chimeric WNV containing ROCV prM-E genes in comparison with the parental WNV_NSW011_ virus showed the exactly opposite characteristics, i.e. enhanced replication in IFN-α/β response-competent cells, increased efficiency in inhibition of JAK-STAT signaling, and higher virulence in mice. Deep sequencing of the recovered viruses identified a number of the same changes from the previously published sequence in all recovered viruses, which we believe to be either mistakes in the original published sequence or changes accumulated during passaging of the ROCV isolate used in this study. Two other identified potential changes, both reflecting mixed virus populations, one at residue 111 in the C gene (mixture of Ala and Ser) and one in NS2A gene (synonymous change at Ala 66 residue) in ROCV/WNV-prME chimeric virus are unlikely to cause profound changes in the virus properties. A mixed population was also identified at residue 89 in the E gene of both, ROCV and WNV/ROCV-prME chimeric virus, likely to produce similar effect on properties of both viruses.

Viruses have evolved many mechanisms to evade the IFN system, blocking at almost every step of the signaling pathway^17^. Several studies, including ours, showed that non-structural proteins of flaviviruses such as DENV, JEV, TBEV, and WNV were responsible for the inhibition of IFN signaling^19^,^20^,^24^,^35^,^36^. Therefore, our findings that ROCV prM-E proteins exhibited strong IFN antagonistic activity was rather unexpected. Interestingly, E protein of JEV was also shown to be involved in inhibition of IFN-α/β response, with a E138K mutation identified in the attenuated strain of JEV shown to result in decreased efficiency in inhibiting IFN-α/β signaling and reduced virulence in mice^25^. The authors suggested that inefficient binding of virions to glycosaminoglycans on cell membrane, viral entry, and/or delayed virus replication exhibited by E138K mutant virus were likely contributing factors to the increased sensitivity to IFN; indicating that a direct interactions of E protein with intracellular cytoplasmic signaling factors were unlikely. In a separate study by Arjona *et al*.^37^, cells treated ectopically with recombinant WNV E protein were shown to have reduced signaling and production of cytokines associated with the dsRNA sensing pathways, mainly inhibition of RIP1 polyubiquitination. The inhibitory effect was dependent on glycosylation, a feature present in both, WNV_NSW2011_ and ROCV, E proteins. Thus, how membrane-sequestered prM and/or E proteins could interact with intracellular cytoplasmic signaling factors remains undetermined. Further studies with ectopically expressed ROCV prM-E proteins are required to determine whether their IFN antagonistic activity is independent of virus replication and to elucidate the exact mechanisms involved.

In summary, we have demonstrated that ROCV prM-E proteins are major contributors to virus virulence in mice and are involved in inhibition of IFN-α/β signaling in infected cells.

## Methods

### Cells lines

Baby hamster kidneys 21 (BHK-21) cells, human embryonic kidney 293 (HEK293T) cells, mouse embryonic fibroblast (MEF) cells, interferon alpha/beta receptor deficient (IFNAR^−/−^) MEF cells, and interferon response factors 3 and 7 deficient (IRF3^−/−^ × IRF7^−/−^) MEF cells were maintained in Dulbecco’s Modified Eagle Medium (DMEM) supplemented with 4–10% of fetal bovine serum (FBS) (Gibco, USA) at 37 °C with 5% of CO_2_. *Aedes albopictus* (C6/36) cells were maintained in Roswell Park Memorial Institute (RPMI) 1640 medium, supplemented with 10% FBS (Gibco, USA) at 28 °C. DMEM and RPMI were supplemented with penicillin (100 U/mL) and streptomycin (100 μg/mL).

### Viruses

*Rocio virus* (SPH 34675 strain) and *West Nile virus* (NSW2011 strain) were maintained in C6/36 cell cultures. The virus titres were determined using plaque assay on BHK cells and expressed as plaque forming units per mL (pfu/mL). The limit of detection (LOD) of this method was 50 PFU/mL. Briefly, 2.5 × 10^5^ cells per well were grown in 6-well plates and infected with ten-fold serial dilutions of the viruses for 1 h at 37 °C; subsequently, 2 mL of 0.375% Low-Melting Point (LMP) agarose in DMEM medium supplemented with 2% FBS were added. Three days post-infection cells were fixed with 4% formaldehyde for 2 hours at room temperature. The LMP agarose medium was removed and the cells were stained with 0.2% crystal violet solution to reveal the plaques.

### Viral RNA purification

The viral RNA was purified from supernatant of infected C6/36 cells using the NucleoSpin RNA Virus kit (MACHEREY-NAGEL, Germany), following the manufacturer’s recommendations.

### Generation of parental and chimeric viruses by CPEC method

The recombinant viruses were generated using CPEC protocol adapted from Setoh *et al*. (2015)^24^. Briefly, RT-PCR fragments spanning the complete viral genome and the flavi-UTR-linker fragment were used to assemble the full-length cDNA. Flavi-UTR-linker fragment contains CMV promoter, conserved first and last 22 nucleotides of viral sequence and hepatitis delta virus ribozyme^23^. Each cDNA fragment contained overlapping ends (21–24 bp) to the adjacent fragments at the 5′and 3′ ends. Fragments of the 5′UTR and 3′UTR regions contained overlapping ends with a flavi-UTR-linker, which allow the generation a circular DNA product and the transcription of the viral RNA in mammalian cells. Primers used to amplify the overlapping cDNA fragments of ROCV, WNV and chimeric viruses are described in the Supplementary Table S1. The DNA bands were purified from the agarose gel using a Wizard SV Gel and PCR Clean-Up System (Promega, USA), following the manufacturer’s recommendations. CPEC reaction was performed with the Q5-Hot Start High Fidelity DNA Polymerase (New England Biolab, UK), using equimolar amounts of all viral genomic fragments and the flavi-UTR-linker fragment. The amplification cycle consisted of an initial denaturation step at 98 °C for 3 minutes, followed by 20 cycles of amplification at 98 °C for 15 seconds, 60 °C for 30 second, and 72 °C for 7 minutes; and a final incubation at 72 °C for 15 minutes. The CPEC reaction product containing the full-length cDNA of the viruses was directly transfected into HEK293T cells (5 × 10^5^ cells per well), using the Lipofectamine LTX reagent (Invitrogen, USA), following the manufacturer’s recommendation. Five days after transfection (passage 0), an aliquot of the supernatant was further passed (passage 1) in HEK293T and/or C6/36 cells. The virus from passage 0 and 1 were titrated by the plaque assay in BHK-21 cells and then stored at −80 °C until use.

### Nucleotide sequencing of chimeric viruses

In order to avoid DNA contamination from CPEC reaction, the viral RNAs were DNase treated before genomic amplification by RT-PCR. To confirm the correct construction of the chimeric viruses, RT-PCR amplicons containing the C-prM and E-NS1 junctions were sequenced by Sanger sequencing at the Australian Genomics Research Facility (AGRF).

### Full genome sequencing of recovered viruses

The full genome of the recovered viruses (all from passage 1) were amplified in three ~5000 bp fragments with ~2000 bp overlap between fragments. The primers used for ROCV/WNV-prME are 1F: AGAAATTCACCTGTGTGAAATTTCACC, 1R: TATTCACGATGGGCGACC, 2F: GCAATCAAAGGAAACCGGG, 2R: GGGCCTCCTTTTGTGTATCC, 3F: ATGAATACTACTACGGTGGGC, 3R: AGATCCTGTGTTTTGCAGCACC. The primers used for WNV/ROCV-prME are 1F: AGCTGACAAACTTAGTAGTGTTTG, 1R: TCCAGTGGGGAAGTCTAAAG, 2F: AGGGTCAGAGAAAGCAACAC, 2R: AGAACACATCCACCCCACTC, 3F: GATGAGTACTGCTATGGAGGG, 3R: AGATCCTGTGTTCTCGCACC. Libraries were prepared using the Nextera XT DNA Sample Preparation Kit (Illumina Inc., San Diego, CA, USA). All libraries were sequenced with an Illumina NextSeq500 platform 2 × with 150 bp High Output v.1 run chemistry. Reads were mapped to their respective genomes using Bowtie 2^38^.

### Kinetics of virus replication in MEF cells

The kinetics of virus replication was analyzed in WT MEF and IFNAR^−/−^ MEF cells. Briefly, 2.5 × 10^5^ cells per well were grown in 6-well plates and infected (MOI = 0.1) with the viruses for 1 h at 37 °C; subsequently the monolayer was washed twice with warm DMEM and then 2 mL of DMEM supplemented with 5% FBS were added. The supernatants of the cells were harvested at 0, 24, 48 and 72 hours post infection (p.i). The viral titer was determined by plaque assay on BHK-21 cells. Three independent experiments were performed.

### Analysis of inhibition of IFN signaling

IRF3^−/−^ × IRF7^−/−^ MEF cells were grown in 6-well plates and infected with the viruses (MOI = 0.1) for 1 h at 37 °C. Cells were washed twice with PBS and then 2 mL of DMEM supplemented with 5% FBS were added. Forty-eight hours after infection, the media was removed and the cells were washed twice with PBS and then treated with 10,000 IU of mouse IFN-β (R&D systems) for 30 minutes at 37 °C. The cells were then fixed with 4% formaldehyde and permeabilized with ice-cold 100% methanol and resuspended in PBS/T before staining with either mouse monoclonal antibody against WNV E (anti-WNV E - 3.91D) and NS1 (anti-WNV NS1–4G4) protein or a polyclonal antibody against ROCV. Cells were also stained with anti-pSTAT1 monoclonal antibody (Cell Signaling Technologies) and analyzed by flow cytometry.

### Virulence in mice

All animal procedures had received prior approval from the University of Queensland Animal Ethics Committee in accordance with the guidelines for animal experimentation as set out by the National Health and Medical Research Council, Australia. Six week-old C57BL6 female mice (purchased from Animal Resources Centre) were infected via the intraperitoneal route with viruses at a dose of 2.6 × 10^4^ pfu. This viral dose was determined in pilot experiments as lethal for ROCV, but non-lethal for WNV_NSW2011_ in mice of this age. Ten mice per group were infected with each virus. Infected animals were monitored daily for 21 days post-infection, and at the first signs of encephalitis (severe hunching, lethargy, eye closure, severe twitching or hind-leg flaccid paralysis) were immediately culled.

### Statistical analysis

The data were analyzed using GraphPad Prism 7.0 software (La Jolla, CA, USA). Parametric one-way ANOVA test was used to compare within groups and Turkey’s Post Hoc multiple range test was used to find the difference between the groups at level p < 0.05. The level of statistical significance was set at 95% (p = 0.05). Survival curves were analyzed using the Log-rank (Mantel-Cox) test.

## Additional Information

**How to cite this article**: Amarilla, A. A. *et al*. Chimeric viruses between Rocio and West Nile: the role for Rocio prM-E proteins in virulence and inhibition of interferon-a/b signaling. *Sci. Rep.* **7**, 44642; doi: 10.1038/srep44642 (2017).

**Publisher's note:** Springer Nature remains neutral with regard to jurisdictional claims in published maps and institutional affiliations.

## Supplementary Material

Supplementary Table S1

Supplementary Table S2

## References

[b1] Roby J. A., Setoh Y. X., Hall R. A. & Khromykh A. A. Post-translational regulation and modifications of flavivirus structural proteins. J Gen Virol 96, 1551–1569, doi: 10.1099/vir.0.000097 (2015). 25711963

[b2] Lindenbach B., Murray C., Thiel H. & Rice C. Flaviviridae. Sixth edn, Vol. One, 1101–51 (Lippincott Williams & Wilkins Wolters Kluwer Busines 2013).

[b3] Medeiros D., Nunes M., Vasconcelos P., Chang G. & Kuno G. Complete genome characterization of Rocio virus (Flavivirus: Flaviviridae), a Brazilian flavivirus isolated from a fatal case of encephalitis during an epidemic in Sao Paulo state. J Gen Virol 88, 2237–2246 (2007). 17622628 10.1099/vir.0.82883-0

[b4] de Souza Lopes O., de Abreu Sacchetta L., Francy D. B., Jakob W. L. & Calisher C. H. Emergence of a new arbovirus disease in Brazil. III. Isolation of Rocio virus from Psorophora Ferox (Humboldt, 1819). Am J Epidemiol 113, 122–125 (1981). 6110335 10.1093/oxfordjournals.aje.a113075

[b5] de Souza Lopes O., Coimbra T. L., de Abreu Sacchetta L. & Calisher C. H. Emergence of a new arbovirus disease in Brazil. I. Isolation and characterization of the etiologic agent, Rocio virus. Am J Epidemiol 107, 444–449 (1978). 665659 10.1093/oxfordjournals.aje.a112563

[b6] de Souza Lopes O., de Abreu Sacchetta L., Coimbra T. L., Pinto G. H. & Glasser C. M. Emergence of a new arbovirus disease in Brazil. II. Epidemiologic studies on 1975 epidemic. Am J Epidemiol 108, 394–401 (1978). 727209 10.1093/oxfordjournals.aje.a112637

[b7] Straatmann A. . [Serological evidence of the circulation of the Rocio arbovirus (Flaviviridae) in Bahia]. Rev Soc Bras Med Trop 30, 511–515 (1997). 9463199 10.1590/s0037-86821997000600012

[b8] Casseb A. R. . Seroprevalence of flaviviruses antibodies in water buffaloes (Bubalus bubalis) in Brazilian Amazon. J Venom Anim Toxins Incl Trop Dis 20, 9, doi: 10.1186/1678-9199-20-9 (2014). 24666635 PMC3974233

[b9] Pauvolid-Corrêa A. . Serological evidence of widespread circulation of West Nile virus and other flaviviruses in equines of the Pantanal, Brazil. PLoS Negl Trop Dis 8, e2706, doi: 10.1371/journal.pntd.0002706 (2014). 24551266 PMC3923745

[b10] Romano-Lieber N. S. & Iversson L. B. [Serological survey on arbovirus infection in residents of an ecological reserve]. Rev Saude Publica 34, 236–242 (2000). 10920445 10.1590/s0034-89102000000300005

[b11] Silva J. R. . A Saint Louis encephalitis and Rocio virus serosurvey in Brazilian horses. Rev Soc Bras Med Trop 47, 414–417 (2014). 25229279 10.1590/0037-8682-0117-2014

[b12] Tiriba A. C. . [Primary human epidemic encephalitis induced by Arbovirus found at the sea shore south of the State of São Paulo. Clinical study in an emergency hospital]. AMB Rev Assoc Med Bras 22, 415–420 (1976). 1087985

[b13] Rosemberg S. Neuropathology of S. Paulo south coast epidemic encephalitis (Rocio flavivurus). J Neurol Sci 45, 1–12 (1980). 7359160 10.1016/s0022-510x(80)80001-3

[b14] de Barros V. E. . An experimental model of meningoencephalomyelitis by Rocio flavivirus in BALB/c mice: inflammatory response, cytokine production, and histopathology. Am J Trop Med Hyg 85, 363–373, doi: 85/2/363[pii]10.4269/ajtmh.2011.10-0246 (2011). 21813860 10.4269/ajtmh.2011.10-0246 PMC3144838

[b15] Randall R. E. & Goodbourn S. Interferons and viruses: an interplay between induction, signalling, antiviral responses and virus countermeasures. J Gen Virol 89, 1–47, doi: 10.1099/vir.0.83391-0 (2008). 18089727

[b16] Platanias L. C. Mechanisms of type-I- and type-II-interferon-mediated signalling. Nat Rev Immunol 5, 375–386, doi: 10.1038/nri1604 (2005). 15864272

[b17] Coccia E. M. & Battistini A. Early IFN type I response: Learning from microbial evasion strategies. Semin Immunol 27, 85–101, doi: 10.1016/j.smim.2015.03.005 (2015). 25869307 PMC7129383

[b18] Sen G. C. Viruses and interferons. Annu Rev Microbiol 55, 255–281, doi: 10.1146/annurev.micro.55.1.255 (2001). 11544356

[b19] Best S. M. . Inhibition of interferon-stimulated JAK-STAT signaling by a tick-borne flavivirus and identification of NS5 as an interferon antagonist. J Virol 79, 12828–12839, doi: 10.1128/JVI.79.20.12828-12839.2005 (2005). 16188985 PMC1235813

[b20] Liu W. . Inhibition of interferon signaling by the New York 99 strain and Kunjin subtype of West Nile virus involves blockage of STAT1 and STAT2 activation by nonstructural proteins. J Virol 79, 1934–1942, doi: 79/3/1934 [pii]10.1128/JVI.79.3.1934-1942.2005 (2005). 15650219 10.1128/JVI.79.3.1934-1942.2005 PMC544092

[b21] Lin R. J., Chang B. L., Yu H. P., Liao C. L. & Lin Y. L. Blocking of interferon-induced Jak-Stat signaling by Japanese encephalitis virus NS5 through a protein tyrosine phosphatase-mediated mechanism. J Virol 80, 5908–5918, doi: 10.1128/JVI.02714-05 (2006). 16731929 PMC1472572

[b22] Muñoz-Jordan J., Sánchez-Burgos G., Laurent-Rolle M. & García-Sastre A. Inhibition of interferon signaling by dengue virus. Proc Natl Acad Sci USA 100, 14333–14338, doi: 2335168100 [pii]10.1073/pnas.2335168100 (2003). 14612562 10.1073/pnas.2335168100 PMC283592

[b23] Daffis S. . The naturally attenuated Kunjin strain of West Nile virus shows enhanced sensitivity to the host type I interferon response. J Virol 85, 5664–5668, doi: 10.1128/JVI.00232-11 (2011). 21411525 PMC3094947

[b24] Laurent-Rolle M. . The NS5 protein of the virulent West Nile virus NY99 strain is a potent antagonist of type I interferon-mediated JAK-STAT signaling. J Virol 84, 3503–3515, doi: 10.1128/JVI.01161-09 (2010). 20106931 PMC2838099

[b25] Liang J. J., Liao C. L., Liao J. T., Lee Y. L. & Lin Y. L. A Japanese encephalitis virus vaccine candidate strain is attenuated by decreasing its interferon antagonistic ability. Vaccine 27, 2746–2754, doi: 10.1016/j.vaccine.2009.03.007 (2009). 19366580

[b26] Setoh Y. X. . Systematic analysis of viral genes responsible for differential virulence between American and Australian West Nile virus strains. The Journal of general virology 96, 1297–1308, doi: 10.1099/vir.0.000069 (2015). 25626681

[b27] Edmonds J. . *A* novel bacterium-free method for generation of flavivirus infectious DNA by circular polymerase extension reaction allows accurate recapitulation of viral heterogeneity. J Virol 87, 2367–2372, doi: 10.1128/JVI.03162-12 (2013). 23236063 PMC3571472

[b28] Quan J. & Tian J. Circular polymerase extension cloning of complex gene libraries and pathways. PLoS One 4, e6441, doi: 10.1371/journal.pone.0006441 (2009). 19649325 PMC2713398

[b29] Frost M. J. . Characterization of virulent West Nile virus Kunjin strain, Australia, 2011. Emerg Infect Dis 18, 792–800, doi: 10.3201/eid1805.111720 (2012). 22516173 PMC3358055

[b30] Chambers T. J., Nestorowicz A., Mason P. W. & Rice C. M. Yellow fever/Japanese encephalitis chimeric viruses: construction and biological properties. J Virol 73, 3095–3101 (1999). 10074160 10.1128/jvi.73.4.3095-3101.1999 PMC104070

[b31] Guirakhoo F. . Recombinant chimeric yellow fever-dengue type 2 virus is immunogenic and protective in nonhuman primates. J Virol 74, 5477–5485 (2000). 10823852 10.1128/jvi.74.12.5477-5485.2000 PMC112032

[b32] Pletnev A. G., Bray M., Huggins J. & Lai C. J. Construction and characterization of chimeric tick-borne encephalitis/dengue type 4 viruses. Proc Natl Acad Sci USA 89, 10532–10536 (1992). 1438242 10.1073/pnas.89.21.10532 PMC50373

[b33] Pletnev A. G. & Men R. Attenuation of the Langat tick-borne flavivirus by chimerization with mosquito-borne flavivirus dengue type 4. Proc Natl Acad Sci USA 95, 1746–1751 (1998). 9465088 10.1073/pnas.95.4.1746 PMC19176

[b34] Arroyo J. . ChimeriVax-West Nile virus live-attenuated vaccine: preclinical evaluation of safety, immunogenicity, and efficacy. J Virol 78, 12497–12507, doi: 10.1128/JVI.78.22.12497-12507.2004 (2004). 15507637 PMC525070

[b35] Lin R. J., Liao C. L., Lin E. & Lin Y. L. Blocking of the alpha interferon-induced Jak-Stat signaling pathway by Japanese encephalitis virus infection. J Virol 78, 9285–9294, doi: 10.1128/JVI.78.17.9285-9294.2004 (2004). 15308723 PMC506928

[b36] Muñoz-Jordán J. . Inhibition of alpha/beta interferon signaling by the NS4B protein of flaviviruses. J Virol 79, 8004–8013, doi: 79/13/8004[pii] 10.1128/JVI.79.13.8004-8013.2005 (2005). 15956546 PMC1143737

[b37] Arjona A. . West Nile virus envelope protein inhibits dsRNA-induced innate immune responses. Journal of immunology 179, 8403–8409 (2007). 10.4049/jimmunol.179.12.8403 18056386

[b38] Langmead B. & Salzberg S. L. Fast gapped-read alignment with Bowtie 2. Nat Methods 9, 357–359, doi: 10.1038/nmeth.1923 (2012). 22388286 10.1038/nmeth.1923 PMC3322381

