# Supplementary material for: Chimeric viruses between Rocio and West Nile: the role for Rocio prM-E proteins in virulence and inhibition of interferon-α/β signaling
Source: Sci Rep. 2017 Mar 20;7:44642. doi: 10.1038/srep44642 (PMC5357910; doi:10.1038/srep44642)
Supplement: Supplementary Table S1 [file srep44642-s1.pdf]

Supplementary Information

Chimeric viruses between Rocio and West Nile: the role for Rocio prM-E proteins in virulence and inhibition of interferon-α/β signaling

Alberto A Amarilla[1¶], Yin X Setoh[2¶], Parthiban Periasamy[2], Nias Y Peng[2], Gabor Pali[2], Luiz T Figueiredo[3], Alexander A Khromykh[2*&], Victor H Aquino[1*&]

[1]Laboratory of Virology, Department of Clinical Analyses, Toxicology and Food Sciences, School of Pharmaceutical Sciences of Ribeirao Preto, University of Sao Paulo, Ribeirao Preto, Sao Paulo, Brazil.

[2]Australian Infectious Diseases Research Centre, School of Chemistry and Molecular Biosciences, The University of Queensland, St Lucia, Queensland, 4072, QLD, Australia.

[3]Virology Research Center, School of Medicine of Ribeirao Preto, University of de Sao Paulo, Ribeirao Preto, Sao Paulo, Brazil.

* Corresponding authors: alexander.khromykh@uq.edu.au (AAK) and vhugo@fcfrp.usp.br (VHA).

[¶]These authors contributed equally and should be considered as joint first authors.

[&]These authors contributed equally and should be considered as joint senior authors

Supplementary Table 1. **Primers used to amplify the overlapping cDNA fragments of ROCV, WNV and chimeric viruses.**

CPEC generated	Amplicon name	Primer name	Sequences (5' ---> 3')	Amplicon Size (bp)	Tm	GC (%)	Size
ROCV	ROCV-Linker	ROCV-Linker-F	**CTGGTGCTGCAAAACACAGGATCT**GGGTCGGCATGGCATCTCCACCTC	1032	61	50	24
		ROCV-Linker-R	**CTGTTGGTGAAATTTCACACAGGTGAATTTCT**CGGTTCACTAAACGAGCTCTGCTTATATAGAC		61	38	32
	5'UTR-CprM-E	ROCV-5'UTR-F	AGAAATTCACCTGTGTGAAATTTCACCAACAG	2445	61	38	32
		ROCV-E-R	GGACGTTCATGGCCAAGAAGAGAAG		61	52	25
	NS1	ROCV-NS1-F	CTTCTCTTCTTGGCCATGAACGTCC	1119	61	52	25
		ROCV-NS1-R	CTGGAACCTGCAGATGTCGTTTCC		61	54	24
	NS2AB	ROCV-NS2A-F	GGAAACGACATCTGCAGGTTCCAG	1093	61	54	24
		ROCV-NS2B-R	CTGGAAGGTCCCACAGAACACCAC		62	58	24
	NS3	ROCV-NS3-F	GTGGTGTTCTGTGGGACCTTCCAG	1853	62	58	24
		ROCV-NS3-R	CTTCCCCGCCGCAAATTCTTTG		61	55	22
	NS4AB	ROCV-NS4A-F	CAAAGAATTTGCGGCGGGGAAG	1237	61	55	22
		ROCV-NS4B-R	CTTCACTTTCGGAGCTTCCAGGTTC		61	52	25
	NS5	ROCV-NS5-F	GAACCTGGAAGCTCCGAAAGTGAAG	2758	61	52	25
		ROCV-NS5-R	CTGGGCTGTTCAGTCTTACAGAACTCC		61	52	27
	3'UTR	ROCV-3'UTR-F	GGAGTTCTGTAAGACTGAACAGCCCAG	436	61	52	27
		ROCV-3'UTR-R	AGATCCTGTGTTTTGCAGCACCAG		61	50	24
ROCV/WNV-prME	5'UTR-C	ROCV-5'UTR-F	AGAAATTCACCTGTGTGAAATTTCACCAACAG	473	61	38	32
		ROCV/WNV (WNV	CTTCCCTTGGAAGTTGGAGAGAGTGACAGCCATTGATCCAGTCACAAGCG		73	52	50
	prM-E	WNV-prM-F	GTCACTCTCTCCAACTTCCAAGGGAAG	2004	61	52	27
		WNV-E-R	AGCATGCACGTTCACGGAAAG		60	52	21
	NS1	ROCV/WNV (ROC	CTTTCCGTGAACGTGCATGCTGACACAGGATGCGCGATTGAC	1110	72	55	42
		ROCV-NS1-R	CTGGAACCTGCAGATGTCGTTTCC		61	54	24
	NS2AB	ROCV-NS2A-F	GGAAACGACATCTGCAGGTTCCAG	1093	61	54	24
		ROCV-NS2B-R	CTGGAAGGTCCCACAGAACACCAC		62	58	24
	NS3A	ROCV-NS3-F	GTGGTGTTCTGTGGGACCTTCCAG	760	62	58	24
		ROCV-NS3-A-R	CCGGAGTGTTCTGCTTTGACGG		61	59	22
	NS3B	ROCV-NS3-A-F	CCGTCAAAGCAGAACACTCCGG	1115	61	59	22
		ROCV-NS3-R	CTTCCCCGCCGCAAATTCTTTG		61	55	22
	NS4AB	ROCV-NS4A-F	CAAAGAATTTGCGGCGGGGAAG	1237	61	55	22
		ROCV-NS4B-R	CTTCACTTTCGGAGCTTCCAGGTTC		61	52	25
	NS5	ROCV-NS5-F	GAACCTGGAAGCTCCGAAAGTGAAG	2758	61	52	25
		ROCV-NS5-R	CTGGGCTGTTCAGTCTTACAGAACTCC		61	52	27
	3'UTR	ROCV-3'UTR-F	GGAGTTCTGTAAGACTGAACAGCCCAG	436	61	52	27
		ROCV-3'UTR-R	AGATCCTGTGTTTTGCAGCACCAG		61	50	24
	ROCV-Linker	ROCV-Linker-F	**CTGGTGCTGCAAAACACAGGATCT**GGGTCGGCATGGCATCTCCACCTC	1032	61	50	24
		ROCV-Linker-R	**CTGTTGGTGAAATTTCACACAGGTGAATTTCT**CGGTTCACTAAACGAGCTCTGCTTATATAGAC		61	38	32
WNV/ROCV-prME	5'UTR-C	WNV-5'UTR-F	AGTAGTTCGCCTGTGTGAGCTG	448	60	55	22
		WNV-C-R	TGCTCCCACGCCAGCAATCAG		**63**	62	21
	prM-E	WNV/ROCV (ROC	CTGATTGCTGGCGTGGGAGCACTGCGCCTGGGGACATATCAAG	2055	75	60	43
		WNV/ROCV (ROC	CCGACTTATATCTATGGCACATCCAGTGTCGGCATGGACGTTCATGGCCAAGAAGAGAAG		73	50	60
	NS1-NS2AB	WNV-NS1-F	GACACTGGATGTGCCATAGATATAAGTCGG	2142	61	47	30
		WNV-NS2B-R	CTCCTCTCTTTGTGTATTGGAGAGTTATC		57	41	29
	NS3	WNV-NS3-F	GATAACTCTCCAATACACAAAGAGAGGAG	1875	57	41	29
		WNV-NS3-R	CCTGAGGCGAAGTCTTTGAA		55	50	20
	NS4AB	WNV-NS4A-F	TTCAAAGACTTCGCCTCAGG	1256	55	50	20
		WNV-NS4B-R	CGTCCTTTTGCCCCACCTC		59	63	19
	NS5	WNV-NS5-F	GAGGTGGGGCAAAAGGACG	2712	59	63	19
		WNV-NS5-R	CTGTATCCTCAACAAATGTCGTGTCTTC		59	43	28
	3'UTR	WNV-3'UTR-F	GAAGACACGACATTTGTTGAGGATACAG	664	59	43	28
		WNV-3'UTR-R	TCCTGTGTTCTCGCACCAC		58	58	19
	WNV-Linker	WNV-Linker-F	GTGGTGCGAGAACACAGGA	1076	58	58	19
		WNV-Linker-R	CAGCTCACACAGGCGAACTACT		60	55	22

ROCV-Linker-F: containig last nucleotides from ROCV (Bold sequences) and first nucleotides of HDVr sequences (underlined letters)

ROCV-Linker-R: containig first nucleotides from ROCV (Bold sequences) and last nucleotides of CMV promotor sequences (underlined letters)
