# Supplementary material for: Chimeric viruses between Rocio and West Nile: the role for Rocio prM-E proteins in virulence and inhibition of interferon-α/β signaling
Source: Sci Rep. 2017 Mar 20;7:44642. doi: 10.1038/srep44642 (PMC5357910; doi:10.1038/srep44642)
Supplement: Supplementary Table S2 [file srep44642-s2.pdf]

Supplementary Information

Chimeric viruses between Rocio and West Nile: the role for Rocio prM-E proteins in virulence and inhibition of interferon-α/β signaling

Alberto A Amarilla[1¶], Yin X Setoh[2¶], Parthiban Periasamy[2], Nias Y Peng[2], Gabor Pali[2], Luiz T Figueiredo[3], Alexander A Khromykh[2*&], Victor H Aquino[1*&]

[1]Laboratory of Virology, Department of Clinical Analyses, Toxicology and Food Sciences, School of Pharmaceutical Sciences of Ribeirao Preto, University of Sao Paulo, Ribeirao Preto, Sao Paulo, Brazil.

[2]Australian Infectious Diseases Research Centre, School of Chemistry and Molecular Biosciences, The University of Queensland, St Lucia, Queensland, 4072, QLD, Australia.

[3]Virology Research Center, School of Medicine of Ribeirao Preto, University of de Sao Paulo, Ribeirao Preto, Sao Paulo, Brazil.

* Corresponding authors: alexander.khromykh@uq.edu.au (AAK) and vhugo@fcfrp.usp.br (VHA).

[¶]These authors contributed equally and should be considered as joint first authors.

[&]These authors contributed equally and should be considered as joint senior authors

Supplementary Table 1. **Deep sequencing analysis of the recovered ROCV and respective chimeric viruses (ROCV/WNV-prME and WNV/ROCV-prME).**

Residues denoted in green correspond to the differences found between recovered ROCV and respective chimeric viruses.

Residue A111 and A66 denoted in red in C and NS2A genes respectively, correspond to the differences between the recovered ROCV and ROCV/WNV-prME.

The substitution, N89, in E gene denoted in blue contains mixed bases in both recovered ROCV and WNV/ROCV-prME, however, the predominant population is the same in both viruses and this is not a glycosylation site.

ROCV

C	prM	E	NS2A	NS3	NS4A	NS5	3' UTR
A111 (GCG) 98% G	N47K (AAT -> AAA) 99% A	N89 (AAC) 30% A	A66 (GCC) 98% C	S157S (TCC -> TCA) 97% A	A134A (GCC -> GCA) 96% A, 3% C	A71V (GCT -> GTT) 97% T, 2% G	Nt 27 (G -> A) 96% A
		N89S (AAC -> AGC) 68% G		H158S (CAC -> AGC) 97% A & 98% G		I123I (ATT -> ATC) 98% C	Nt 121 (A -> T) 96% T, 2% G
		Y235N (TAC -> AAC) 98% A		S225T (AGT -> ACT) 98% C		Q337Q (CAA -> CAG) 98% G	Nt 141 (T -> A) 99% A
		V247A (GTT -> GCT) 98% C		E575E (GAA -> GAG) 98% G		A373V (GCG -> GTG) 96% T, 3% G	Nt 194 (T -> A) 99% A
		E261A (GAA -> GCA) 98% C				F435C (TTT -> CTC) 97% C & 97% C	Nt 200 (T -> C) 99% C
		I264T (ATT -> ACT) 99% C				I452I (ATT -> ATC) 98% C	Nt 219 (A -> C) 99% C
		V265A (GTT -> GCT) 98% C				T660S (ACT -> AGT) 99% G	
		L279L (TTA -> CTA) 99% C				P661R (CCG -> CGG) 99% G	
		K409K (AAA -> AAG) 98% G					

ROCV/WNV-prME

C	prM	E	NS2A	NS3	NS4A	NS5	3' UTR
A111S (GCG -> TCG) 70% T, 27% G			A66A (GCC -> GCT) 62% T, 36% C	S157S (TCC -> TCA) 97% A	A134A (GCC -> GCA) 99% A	A71V (GCT -> GTT) 97% T	Nt 27 (G -> A) 99% A
				H158S (CAC -> AGC) 98% A & 99% G		I123I (ATT -> ATC) 99% C	Nt 121 (A -> T) 98% T
				S225T (AGT -> ACT) 99% C		Q337Q (CAA -> CAG) 99% G	Nt 141 (T -> A) 99% A
				E575E (GAA -> GAG) 99% G		A373V (GCG -> GTG) 96% T	Nt 194 (T -> A) 99% A
						F435C (TTT -> CTC) 98% C1 & 99% C3	Nt 200 (T -> C) 99% C
						I452I (ATT -> ATC) 98% C	Nt 219 (A -> C) 98% C
						T660S (ACT -> AGT) 99% G	
						P661R (CCG -> CGG) 99% G	

WNV/ROCV-prME

C	prM	E	NS2A	NS3	NS4A	NS5	3' UTR
	N47K (AAT -> AAA) 98% A	N89 (AAC) 46% A					
		N89S (AAC -> AGC) 52% G					
		Y235N (TAC -> AAC) 96% A					
		V247A (GTT -> GCT) 99% C					
		E261A (GAA -> GCA) 98% C					
		I264T (ATT -> ACT) 99% C					
		V265A (GTT -> GCT) 99% C					
		L279L (TTA -> CTA) 99% C					
		K409K (AAA -> AAG) 99% G					
